# How Do Antihypertensive Drugs Work? Insights from Studies of the Renal Regulation of Arterial Blood Pressure

**DOI:** 10.3389/fphys.2016.00320

**Published:** 2016-07-29

**Authors:** Holly Digne-Malcolm, Matthew C. Frise, Keith L. Dorrington

**Affiliations:** ^1^Medical Sciences Division, University of OxfordOxford, UK; ^2^Department of Physiology, Anatomy and Genetics, University of OxfordOxford, UK; ^3^Nuffield Department of Anaesthetics, John Radcliffe HospitalOxford, UK

**Keywords:** antihypertensive drugs, renal circulation, hypertension, diuretics, vasodilator agents

## Abstract

Though antihypertensive drugs have been in use for many decades, the mechanisms by which they act chronically to reduce blood pressure remain unclear. Over long periods, mean arterial blood pressure must match the perfusion pressure necessary for the kidney to achieve its role in eliminating the daily intake of salt and water. It follows that the kidney is the most likely target for the action of most effective antihypertensive agents used chronically in clinical practice today. Here we review the long-term renal actions of antihypertensive agents in human studies and find three different mechanisms of action for the drugs investigated. (i) Selective vasodilatation of the renal afferent arteriole (prazosin, indoramin, clonidine, moxonidine, α-methyldopa, some Ca^++^-channel blockers, angiotensin-receptor blockers, atenolol, metoprolol, bisoprolol, labetolol, hydrochlorothiazide, and furosemide). (ii) Inhibition of tubular solute reabsorption (propranolol, nadolol, oxprenolol, and indapamide). (iii) A combination of these first two mechanisms (amlodipine, nifedipine and ACE-inhibitors). These findings provide insights into the actions of antihypertensive drugs, and challenge misconceptions about the mechanisms underlying the therapeutic efficacy of many of the agents.

## Introduction

### Why is this topic currently especially important?

The long-term regulation of systemic arterial blood pressure is a topic beset with a remarkable degree of controversy. Many textbooks teach that systemic vascular resistance (SVR) is the primary factor in this long-term regulation (Pocock and Richards, 2006; Levick, 2010; Beevers et al., 2014), a view that continues to be aired in peer-reviewed scientific journals (Averina et al., 2012; Pettersen et al., 2014; Kurtz et al., 2015; Joyner et al., 2016). Some texts highlight the role of the kidney in setting arterial blood pressure, emphasizing its role in determining the volume of fluid in the circulation (Boron and Boulpaep, 2012; Mohrman and Heller, 2014). Sometimes these discordant viewpoints are presented one following another without resolution (Steddon et al., 2007). The confusion associated with this topic is important because extensive resources are directed toward the diagnosis and treatment of hypertension, since sustained elevation of arterial blood pressure makes a very considerable and geographically variable contribution to the global burden of mortality from non-communicable diseases (Forouzanfar et al., 2015). Thus, for example, a recent comparison between the USA, Canada and England has shown high prevalences of hypertension, a strong relationship between indicators of hypertension and both stroke mortality and ischemic heart disease mortality, and rather dramatic differences between these three countries (Joffres et al., 2013). Recognition and management of hypertension appear to be strongest in Canada, followed by the USA, followed by particularly alarming figures for England. Given that the therapeutic activity of antihypertensive drugs is to a large extent empirical and may itself be associated with a variety of adverse effects, clear targeting of relevant tissue components in whatever organ system does regulate arterial pressure over long periods of time, would be highly desirable.

That modulation of SVR is a major factor in determining the long-term setting of arterial pressure is widely inferred from its precise relationship with mean arterial pressure (MAP) via what we can regard as Ohm's Law for the systemic circulation:

(1) $$(\text{MAP}-\text{CVP})=\dot{\text{Q}}\times \text{SVR},$$

where CVP is central venous pressure and $\overset{\cdot }{\text{Q}}$ is cardiac output. Moreover, it is a common observation that $\overset{\cdot }{\text{Q}}$ is often largely unchanged in hypertension, whilst CVP is small in relation to MAP, and also often largely unchanging. Equation (1) inclines us to think in terms of MAP as being almost mathematically proportional to SVR, and hence to consider SVR as being a major determinant of MAP. The common usage that variables on the left-hand side of an equation are dependent variables, whilst those on the right-hand side are independent variables, has tended to reinforce this emphasis.

An alternative view is that it is more helpful to think of Equation 1 in a rearranged form:

(2) $$\dot{\text{Q}}=(\text{MAP}-\text{CVP})/\text{SVR},$$

where MAP is determined slowly over time by the need for the circulation to be in “mass balance” (a view expanded on below), and SVR is set by many known (and as yet unknown) structural, neural, humoral, and pharmacological factors. In this approach $\overset{\cdot }{\text{Q}}$ (and CVP) are viewed as the dependent variables that are constrained by a need to satisfy this strict physiological relationship, as well as the properties of the heart known as the “Frank-Starling” relationship between $\overset{\cdot }{\text{Q}}$ and CVP. This is the view taken by some authors giving what we may think of as a “descriptive” account of physiological measurements (Hall, 1999; Montani and Van Vliet, 2009; Ivy and Bailey, 2014). It is also the view to which authors using theoretical mathematical models of the circulation have found themselves driven (Guyton, 1980, 1987; Keener and Sneyd, 2009; van den Berg, 2011).

Several associations between SVR and MAP make it difficult for researchers and healthcare workers to form a clear view about cause and effect. First, SVR is indeed typically raised in hypertension—an association that does not necessarily imply direct causation. Secondly, it seems likely that the physiological processes that commonly lead to changes in the kidney and its vasculature, which may be the direct cause of hypertension, concurrently elevate SVR by affecting blood vessels throughout the body. Thirdly, many drugs that act on the kidney to ameliorate hypertension also act on the wider circulation to lower SVR. Fourthly, *short-term* changes in arterial blood pressure are very commonly brought about by changes in SVR, either spontaneously or therapeutically. A truly integrative approach is needed to separate causation from association, and thus the topic provides an outstanding educational challenge as well as a scientific battlefield.

The aim of this paper is both educational and scientific. We give the basis for the view that renal mechanisms regulate arterial blood pressure on a time scale of days to years. We then review what is known about how antihypertensive drugs act upon the kidney to achieve a sustained reduction in arterial pressure. This enables a clear distinction to be drawn between the incidental effects these drugs have elsewhere on the body, which may be harmful or beneficial, and their primary therapeutic renal actions in hypertension.

### Short-term vs. long-term regulation of arterial blood pressure

A considerable variability of arterial blood pressure obtained from one measurement to another over short periods of time is widely observed in patients and healthy individuals, even at rest (Littler et al., 1978; deBoer et al., 1987; Rothwell, 2010). Perhaps more than physicians in any other specialty, anesthetists are familiar with exaggerations of normal changes; it is not uncommon to see the rapid halving or doubling of arterial blood pressure in response to drugs such as anesthetics, vasodilators, and catecholamines, as well as to surgical events. Short-term lability of blood pressure can be thought of as resulting from rapid changes in SVR, vascular compliance, cardiac contractility, heart rate, and (in the presence of bleeding or infusion) circulating volume. The interplay of these factors, when precisely defined, enables MAP to be modeled in what might be regarded as a *closed circulation* (Dorrington and Pandit, 2009; Keener and Sneyd, 2009).

Amidst the “noise” of short-term variations in blood pressure it is easy to lose sight of the constraint imposed upon the circulation that engineers call “mass balance.” Over prolonged periods of time the inputs of salt and water to the circulation must match their output. Since the kidney provides the main regulated route for excretion of salt and water, the pressure in the arterial tree must equilibrate over time with the pressure required by the kidney to perform this ongoing task. Taking this view of an *open circulation* it follows that MAP over prolonged periods is determined by one “final common path”—to use the term Sherrington (Sherrington, 1920) gave to the lower motor neuron of the nervous system—in this case the kidney (Dorrington and Pandit, 2009; Keener and Sneyd, 2009; van den Berg, 2011). This pressure will be a factor not only of the intrinsic structure of the kidney; it will be affected by neural, humoral, and pharmacological regulators of renal function; here we assess this latter group.

It is important to appreciate that what is both approvingly and sometimes dismissively called the “Guyton model” of long-term blood pressure control, following determined advocacy by Arthur Guyton over many years (Guyton, 1980, 1991), is fundamentally an expression of mass balance in the circulation, and might be less controversially named as such. Perhaps a term such as a “physical equilibrium model” would carry less historical baggage for some workers in the field, whilst others continue to see the eponymous attribution as giving due credit to a distinguished physiologist. The equilibrium has probably been depicted in its most simple and accessible analogy by Kimura et al. who depicted arterial blood pressure as the height of liquid in a water tank, from which the only elimination was via an outflow pipe representing the kidney (Kimura et al., 1986). This model was later used to examine the hypotensive actions of different families of drugs in a strikingly perceptive manner (Kimura et al., 1989) and to clarify the sensitivity of high blood pressure to daily sodium excretion (Kimura et al., 1990).

### How can the kidney do the same job at a lower arterial pressure?

Two main ways can be envisaged in which drugs may permit the kidney to continue to eliminate the normal daily intake of salt and water whilst being perfused from an arterial tree at a reduced pressure: either a vascular or a tubular mechanism. Exchange of water and solutes across any epithelium is a function of the “Starling forces,” the hydrostatic and osmotic pressure differences across the epithelium, and the vigor of actively-driven cellular transport mechanisms that act to modify the osmotic pressures. The interrelation between these determinants of transepithelial flux has been widely explored in relation to the kidney (Martino and Earley, 1968; Knox et al., 1983) and can also be demonstrated in other epithelia such as those in the intestine (Mailman, 1982) and the lung (Vejlstrup et al., 1994).

Perhaps the simplest way for a vasoactive drug to permit the kidney to perform its normal handling of salt and water at a reduced MAP is via dilatation of the afferent arteriole leading to the glomerulus. Isolated afferent arteriolar vasodilatation could allow the glomerular capillaries to be perfused at an unchanged pressure in the presence of a low MAP. This could maintain an unchanged glomerular filtration rate (GFR) and an unchanged renal blood flow (RBF), whilst a measurement of renal vascular resistance (if defined as MAP/RBF) would show it to have decreased. Such an intervention would be analogous to the surgical removal of a renal artery stenosis, a classic example of “secondary hypertension,” eliminating an excessive pressure drop across the vessels entering the kidney, so that from the glomerular capillaries onwards nothing is different after the intervention when compared with before the intervention. It will become clear that the actions of several families of drugs converge on this common pathway.

The “non-vascular” route to modifying the pressure at which the kidney operates involves the inhibition of active Na^+^ and other ionic reabsorption from the renal tubule into the peritubular capillaries: a tubular, epithelial, mode of action. Inhibition of the active component of reabsorption has to translate into a readjustment of Starling forces to maintain reabsorption and keep the body in sodium and water balance. This in turn requires a lower hydrostatic pressure in the peritubular capillaries and throughout the renal vascular bed, including the glomerular capillaries. Some antihypertensive drugs are associated with a reduction in GFR and appear to have their main renal action on inhibition of active Na^+^ and water reabsorption, and the consequent resetting of the Starling forces across the tubular epithelium. A review of the literature leads to surprises in this area.

If an antihypertensive can be associated with a fall in GFR, under what circumstances may one be associated with a rise in GFR? In fact, a large family of drugs is capable of bringing about an increase in hydrostatic pressure gradient along the efferent arteriole of the kidney, generated either by an increased resistance in, or a higher blood flow through, these vessels. These drugs achieve marked afferent arteriolar dilatation with a degree of inhibition of active tubular reabsorption, combining a vascular action with a tubular action.

## A historical perspective on methodology

### Measurement of renal hemodynamics in humans

We are fortunate in having two sound, long-standing methods for measuring RBF and GFR in humans. Both rely on measuring “renal clearance.” The clearance of a substance in the blood is the volume of blood from which the substance can be regarded as having been completely eliminated in unit time, and is therefore measured in units such as ml/min. A small derivative of glycine, para-aminohippurate (PAH), is both filtered at the renal glomerulus and secreted in the renal tubule. This almost complete removal of PAH from blood passing through the kidney leads to its renal clearance being a measure of RBF (Chasis et al., 1945). Inulin is a polysaccharide that is filtered at the glomerulus whilst being neither secreted nor absorbed by the renal tubule in any appreciable amount (Shannon and Smith, 1935). It follows that its renal clearance is a measure of GFR, long seen as an indicator of the extent to which the kidney is functioning satisfactorily. Some studies use the clearance of the endogenous molecule creatinine as an approximation to the GFR (Preston et al., 1979), as in clinical practice.

Since GFR is determined in part by glomerular capillary pressure (P_glom_) there has been much interest in attempting to measure P_glom_ and observe changes in these variables associated with essential hypertension and with antihypertensive medication. Unfortunately, direct measurement of P_glom_ has not proved possible in humans, and researchers remain dependent upon the approach of Gómez published over half a century ago (Gomez, 1951). We briefly summarize this approach in the following section, because its limitations need to be taken into account.

### Estimation of glomerular capillary pressure

**Figure 1:**
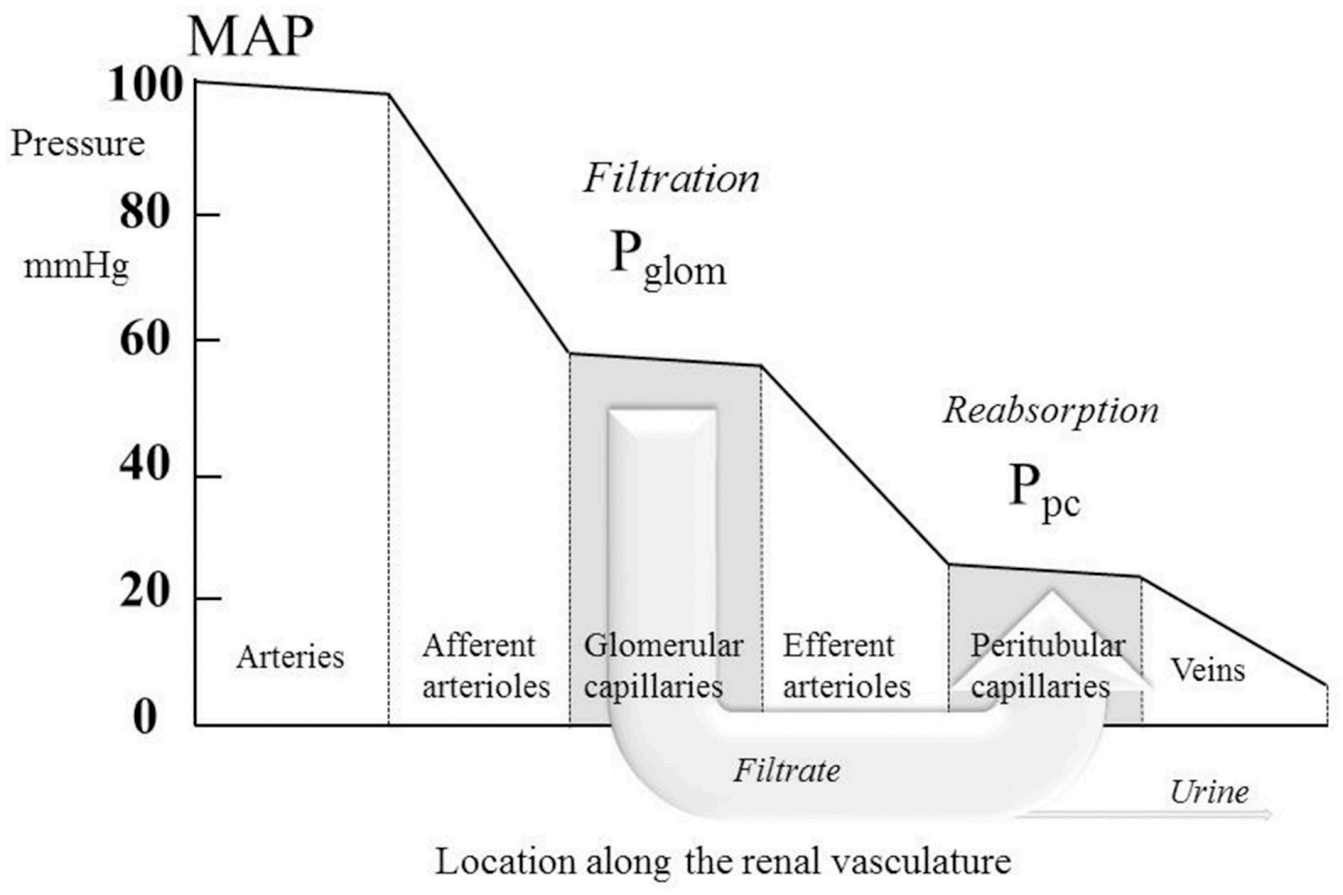
**The profile of hydrostatic pressures along the renal vasculature, showing typical normal values**. MAP, mean arterial pressure; Pglom, glomerular pressure; Ppc, peritubular capillary pressure. The large arrow labeled “Filtrate” is a cartoon representation of the flow of glomerular filtrate, most of which is reabsorbed from the tubule into the peritubular capillaries. The small fraction of the filtrate that is not reabsorbed is depicted by the arrow labeled “Urine.”

Domingo Gómez derived P_glom_ from measured GFR by assuming a “normal” value from the literature of the glomerular permeability coefficient (taking into account body size), which we here write as K, and writing the Starling relationship for glomerular filtration as follows:

(3) $$\text{GFR}=\text{K}({\text{P}}_{\text{glom}}-{\text{P}}_{\text{tub}}-\Pi ),$$

where P_tub_ is the hydrostatic pressure in Bowman's space (and the whole renal tubule) and Π is the oncotic pressure of plasma. P_tub_ is commonly assumed in humans to be similar to that measured directly in other mammals (Ichikawa and Brenner, 1980; Bencsath et al., 1983) and to equal 10–15 mmHg, and Π is calculated from plasma protein concentration using various formulae; estimations of Π are usually close to 25 mmHg (Bencsath et al., 1983). Estimates of P_glom_ are commonly close to 60 mmHg with the net filtration pressure around 60 − 10 − 25 = 25 mmHg. In 22 normotensive humans, Gómez estimated a mean value for P_glom_ of 63 mmHg (derived from Gomez, 1951, Table 1). Figure 1 illustrates the pressure profile in the blood vessels passing through the kidney, based in part on direct measurement of pressures in animal preparations (Carmines et al., 1990).

Of special interest in relation to hypertension is the question of whether a major contributor to the condition is an abnormally elevated afferent arteriolar resistance (R_A_), defined as the resistance of blood vessels between the large arteries and the glomerular capillaries:

(4) $${\text{R}}_{\text{A}}=(\text{MAP}-{\text{P}}_{\text{glom}})/\text{RBF}.$$

The behavior of the efferent arteriole leaving the glomerulus is also of interest because its resistance R_E_ relative to R_A_ plays a role in determining both RBF and P_glom_. Here the analysis meets difficulties; another unknown is the mean pressure in the peritubular capillaries P_pc_, downstream of the efferent arteriole, which is required for a calculation of R_E_:

(5) $${\text{R}}_{\text{E}}=({\text{P}}_{\text{glom}}-{\text{P}}_{\text{pc}})/(\text{RBF}-\text{GFR}).$$

Note that in this equation the flow of blood through the efferent arterioles is now RBF minus the flow of the glomerular filtrate, a flow diverted past the efferent arteriole via the renal tubule; this flow is represented in cartoon form in Figure 1 by the large arrow labeled “*Filtrate*.”

Gómez took the view that the surface area of the peritubular capillaries was likely to be so much greater (by 15–60 fold) than that of the glomerular capillaries that the net filtration pressure across the tubule-capillary barrier would be approximately zero. This can be expressed as:

(6) $${\text{P}}_{\text{pc}}\approx {\text{P}}_{\text{tub}}+\Pi ,$$

yielding pressures in the peritubular capillaries of around 35 mmHg. If Equation (6) is taken as precise, a combination of Equations (3, 5, and 6) then gives:

(7) $${\text{R}}_{\text{E}}=\text{GFR}/(\text{K}(\text{RBF}-\text{GFR})).$$

Some authors prefer to complete the picture of renal vascular behavior by including the venular resistance, defined as:

(8) $${\text{R}}_{\text{V}}=({\text{P}}_{\text{pc}}-{\text{P}}_{\text{v}})/\text{RBF},$$

where P_v_ is the pressure in the renal veins, commonly assumed to be around 10 mmHg. Clearly, there is an assumption here that the urine flow is so small in comparison with RBF that it can be ignored from the calculation of the resistance of the venules. The small fraction of the filtrate is labeled “*Urine*” in Figure 1.

Venular pressure can be measured in humans directly by passing a catheter into the renal veins. In 1970, Lowenstein et al. developed the technique of advancing a 0.9 mm diameter polyethylene catheter from the renal vein (where a typical P_v_ was ~6 mmHg) until it wedged in a smaller vein and recorded a pressure taken to be that of the peritubular capillaries, P_pc_ (Lowenstein et al., 1970). Their estimates of P_pc_ from this “renal vein wedge pressure” varied considerably between normal individuals in the approximate range 20–30 mmHg following a period of 12–16 h of fluid restriction. Higher values were obtained in hypertensives and in all volunteers during diuresis following infusions of saline or mannitol. Using the same technique, Willassen and Ofstad later measured P_pc_ to be ~25 mmHg in fasted healthy volunteers and hypertensives, with values of P_v_ close to 2 mmHg (Willassen and Ofstad, 1980). These data from humans suggest that Gómez's estimate of 35 mmHg for P_pc_ is rather high, and that there may normally be a substantial gradient in Starling forces driving reabsorption into the peritubular capillaries, such that P_pc_ < (P_tub_ + Π) by a substantial number of mmHg. Direct puncture measurements in small mammals have tended to suggest this too. Ichikawa and Brenner, using direct puncture measurements in rats, found that both P_pc_ and P_tub_ were close to 15 mmHg whilst Π was ~25 mmHg, suggesting a net reabsorption Starling pressure between the tubule and peritubular capillary of about 25 mmHg (Ichikawa and Brenner, 1980), a net Starling “force” similar to that driving glomerular filtration. The estimate of R_E_ given by Equation (5) then becomes an underestimate of the true value by of the order of 50%.

### Renal hemodynamic changes observed by gómez in hypertensive patients

Gómez proceeded to examine the magnitudes of R_A_, R_E_, and R_V_ in 22 healthy subjects and in 46 patients with essential hypertension (Gomez, 1951). A striking difference was observed between the two groups. In the healthy subjects, the three vascular resistances were approximately equal. In the hypertensive patients the prominent finding was that R_A_ was five times greater than in the healthy subjects. In 16 of the hypertensive humans, Gómez measured a mean value for P_glom_ of 47 mmHg (his Table 2). Interestingly this is somewhat lower than the value he found for normotensives (63 mmHg).

He went on to draw a conclusion about the relative magnitudes in the hypertensive patients of SVR and R_A_. He noted that R_A_ tended to correlate with SVR in a pattern in which a doubling in SVR was associated with an approximate 5-fold increase in R_A_. This early analysis pointed strongly toward an abnormally intense increase in R_A_ as the defining feature of essential hypertension. It appeared that some mechanism was capable of increasing arteriolar resistance throughout the body, but was unusually potent in its effects on the renal afferent arterioles (Coleman et al., 1975).

The Gómez formulae have been widely used (Preston et al., 1979; Inigo et al., 2001; Delles et al., 2003; Ott et al., 2013). Interestingly, they were derived before the processes of active tubular epithelial transport were elucidated by Koefoed-Johnsen and Ussing (1958). By 1951 it was already recognized that glomerular filtration was a form of passive ultrafiltration driven by Starling forces and that renal tubular uptake was characterized by some form of active “specific absorbing mechanisms for the separate ions” (Krogh, 1946) modulating the ubiquitous Starling forces. In the light of this, the model of Gómez has to be interpreted as subsuming any such active transport within the cautious assumption expressed in Equation (6) of, on average along the tubule, Starling forces across the tubule-capillary wall of no more than a few mmHg. We have noted above a weakness of this assumption and the probability that there may be a considerable gradient of Starling forces of ~25 mmHg driving reabsorption despite the presence of transcellular active solute transport. We now know much detail about active transport processes in the different segments of the renal tubule, in some regions of which there may be low permeability to water and consequently a gradient in Starling forces. It remains striking, however, that a reviewer of fluid transport by epithelia as recently as 2008 should have to write that “despite a voluminous literature, however, there is no clear idea of how it occurs” (Hill, 2008). Both in relation to Gómez's original assumptions in 1951, and the subsequent expansion of our knowledge about active tubular transport, we are left with continued uncertainty about the precision with which R_E_ and R_V_ can be separately obtained in humans using the above formulae.

### Kimura's method of estimating glomerular capillary pressure

An alternative approach to the measurement of R_A_ has been proposed by Kimura et al. (1991b). Thirty hypertensive patients were studied over a 2-week period on a regular sodium diet (intake 223 meq/day) for 1 week and then on a low sodium diet (intake 30 meq/day) for 1 week. Values of MAP measured after each of these weeks were respectively 120 mmHg and 109 mmHg. A renal pressure-natriuresis line (sometimes called a “renal function curve”) was plotted using these two data points and extrapolated to obtain the hypothetical MAP at which sodium elimination would be zero, MAP_o_. These investigators found MAP_o_ to be 107 mmHg, and argued that, since reduction in MAP from its normal value on a regular diet (120 mmHg) to MAP_o_ would result in cessation of renal sodium clearance, MAP − MAP_o_ must equal the net filtration pressure across the glomerular capillaries (P_glom_ − P_tub_ − Π) whilst on the normal diet. For the group of patients as a whole, application of this approach predicted mean values of P_glom_ and R_A_ that were respectively 7% lower and 6% higher than those obtain using Gómez's formulae. An important advantage of the approach of Kimura et al. is that no assumption needs to be made about the value of the glomerular permeability coefficient, K. A significant drawback, however, is the complexity and duration of the experiments required, including the compliance of patients with a low sodium diet for at least 1 week. Comparison of the results from the two approaches suggests that they both provide satisfactory means for estimating P_glom_ and R_A_, but the estimation of R_E_ and R_V_ remains problematic, for reasons discussed above. Rat experiments have shown a fair agreement between values of P_glom_ obtained using the pressure-natriuresis extrapolation and direct puncture of glomerular capillaries (Kimura and Brenner, 1997). As Gómez had found much earlier, Kimura et al. concluded that the hypertension in their patients was characterized by very high values of R_A_ in the presence of relatively normal values of both P_glom_ and R_E_. They subsequently applied their methodology to examining the renal hemodynamic effects of the antihypertensive drug nicardipine (Kimura et al., 1991a).

Despite difficulties estimating P_pc_, R_E_, and R_v_ in human studies, the relationship between GFR and P_glom_ expressed in Equation (3) remains well established, and allows some confidence in concluding that human studies demonstrating a rise or fall in GFR in individuals to whom antihypertensive drugs are administered are experiencing a corresponding rise or fall, respectively, in P_glom_. We make use of this assumption in deducing changes in the hydrostatic pressure profile along the renal vasculature.

### What the pressure-natriuresis line tells us about renal hemodynamics

**Figure 2:**
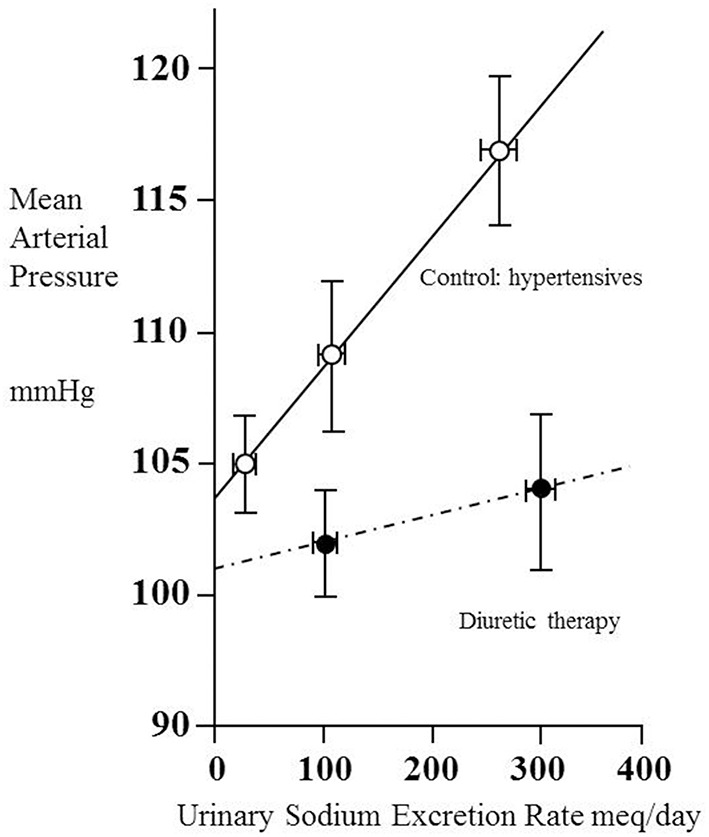
**Pressure–natriuresis lines for eight hypertensive adult patients before (open circles) and after (closed circles) administration of a thiazide diuretic (mefruside 25 mg daily)**. Urinary sodium excretion rate was measured after several periods of 7 days of constant daily sodium intake of between 1 and 18 g NaCl and systemic mean arterial pressure plotted as a function of sodium excretion. Data from Saito and Kimura (1996). Note that the untreated hypertensive patients showed a marked dependence of mean arterial pressure (MAP) on sodium excretion, whilst thiazide administration reduced this dependence and also lowered MAP.

Kimura and colleagues examined the pressure-natriuresis relationship for eight hypertensive patients using exposure to three different values of sodium excretion to assess to what extent they could regard the relationship as linear (Saito and Kimura, 1996). Figure 2 shows the result. Though based only upon three data points (each taken after 1 week on a regulated sodium intake) the relationship appears linear. A striking feature is that MAP increases greatly with sodium intake/excretion over the approximate 10-fold range. Note the intercept at zero sodium excretion of 104 mmHg, similar to the value of 107 mmHg referred to above in relation to another set of (30) hypertensive patients.

We have already noted the concept that this intercept gives information about the net filtration pressure at the glomerulus, implying that, if the intercept were to remain unchanged during administration of a drug, then for any given value of MAP the value of P_glom_ would be the same with or without the drug. Figure 2 contains data obtained from the same patients under the condition of taking the antihypertensive thiazide-like diuretic mefruside. The authors found no statistically significant difference between the intercepts with and without mefruside, whereas the slope of the relationship was changed significantly (*p* < 0.005). Mefruside moved the line toward the horizontal and lowered MAP over the range of sodium intakes.

It is interesting to consider what the significance would have been were a drug like mefruside able to render the line completely horizontal so that MAP became independent of sodium intake. Following the approach of Gómez given earlier, this would imply that the net filtration pressure across the glomerular capillaries (P_glom_ − P_tub_ − Π) was approximately zero and that P_glom_ was close to (P_tub+_ Π), which is commonly taken to equal 35 mmHg. If the imaginary drug were capable of inhibiting all active reabsorption of solutes from the renal tubule, we would then have a situation where the filtered sodium load entering the renal tubule from the glomerulus in a very low GFR (requiring only a small net glomerular filtration pressure) was all lost in the urine!

In this light, it may seem surprising that the normal healthy pressure-natriuresis line is indeed fairly flat, as has been seen in four-data-point plots obtained from dogs (Hall et al., 1980) and using three- or two-data-point plots in healthy humans (Parfrey et al., 1981a,b; Fukuda and Kimura, 2006). Indeed, the flatness of the relationship in health is a remarkable reflection of how the neural, humoral, or intrinsic regulation of the kidney is capable of permitting large changes in the renal elimination of salt *without* there being a large change in MAP. Bie has noted that modest acute body loading with sodium (over 2–3 h) can lead to a rapid substantial change in renal sodium excretion rate without a change in MAP, and that this phenomenon is strong evidence either for a vigorous *macular densa* tubulo-glomerular feedback mechanism, or for a further, as yet unidentified, regulator of renal function (Bie, 2009). Whether the mechanisms regulating these short-term responses are also those acting in the longer term remains to be seen.

After analyzing the effects of different families of drugs on renal hemodynamics in the sections below, we shall be in a position to assess this apparent inconsistency of mefruside yielding a fairly flat pressure-natriuresis line, and also explore those situations in which antihypertensive drugs leave the gradient of the pressure-natriuresis line unchanged, and when they can make it steeper. We shall see that the pressure natriuresis line provides information that could usefully guide therapy if it were to be more widely available and its significance better understood.

### Overview of renal hemodynamic abnormalities that may lead to hypertension

Before surveying the literature for the renal hemodynamic effects of long-term antihypertensive medication it may be helpful to summarize what kind of abnormalities might have the potential to induce hypertension in the first place. We have alluded already to increased pre-glomerular resistance, and noted Gómez's early striking observation of this phenomenon in individuals with essential hypertension (Gomez, 1951), which has been confirmed by others (Kimura et al., 1991b). The possibility of a reduction of the glomerular permeability coefficient K (Equation 3) in some kinds of hypertension appears to lead to high P_glom_ and thereby high MAP (Sanai and Kimura, 1996), and is discussed further below in relation to mesangial cells. Loss of functional nephrons, an inability to modulate the renin-angiotensin-aldosterone system appropriately (Hall et al., 1980), and the various genetic abnormalities of renal tubular sodium uptake (Kurtz et al., 2015) are all material, even though their precise mode of influencing blood pressure may remain uncertain.

## Results from the literature

We surveyed the literature for studies in healthy humans and patients with hypertension in which measurements of arterial blood pressure and renal hemodynamics had been made in the control condition of no drug therapy and following a minimum of 1 week of sustained drug therapy. We included only those in which a statistically significant fall in MAP (or *both* systolic and diastolic pressure) had been found on drug therapy. In the tables below we present statistically significant changes in renal hemodynamic variables or indicate no change. A point of note arises in relation to the overall RVR defined and calculated by most authors as follows:

(9) RVR=MAP/RBF.

In some studies, no statistically significant change in either RBF or RVR was found, despite the fall in MAP. To help with data interpretation, such apparently anomalous findings are indicated in the tables with a footnote to explain the anomaly.

Our on-line search strategy was to use Web of Science™, entering the names of specific agents or drug classes, and linking these, in turn, with the terms “renal hemodynamics” (in UK and USA spelling), “glomerular filtration rate,” “renal blood flow,” and “renal plasma flow.” We surveyed titles and abstracts to select human studies on both healthy volunteers and patients with hypertension that was found not to be attributable to secondary (known) causes. Reference lists from publications identified in this way were surveyed for further relevant publications.

### Vasodilators

**Figure 3:**
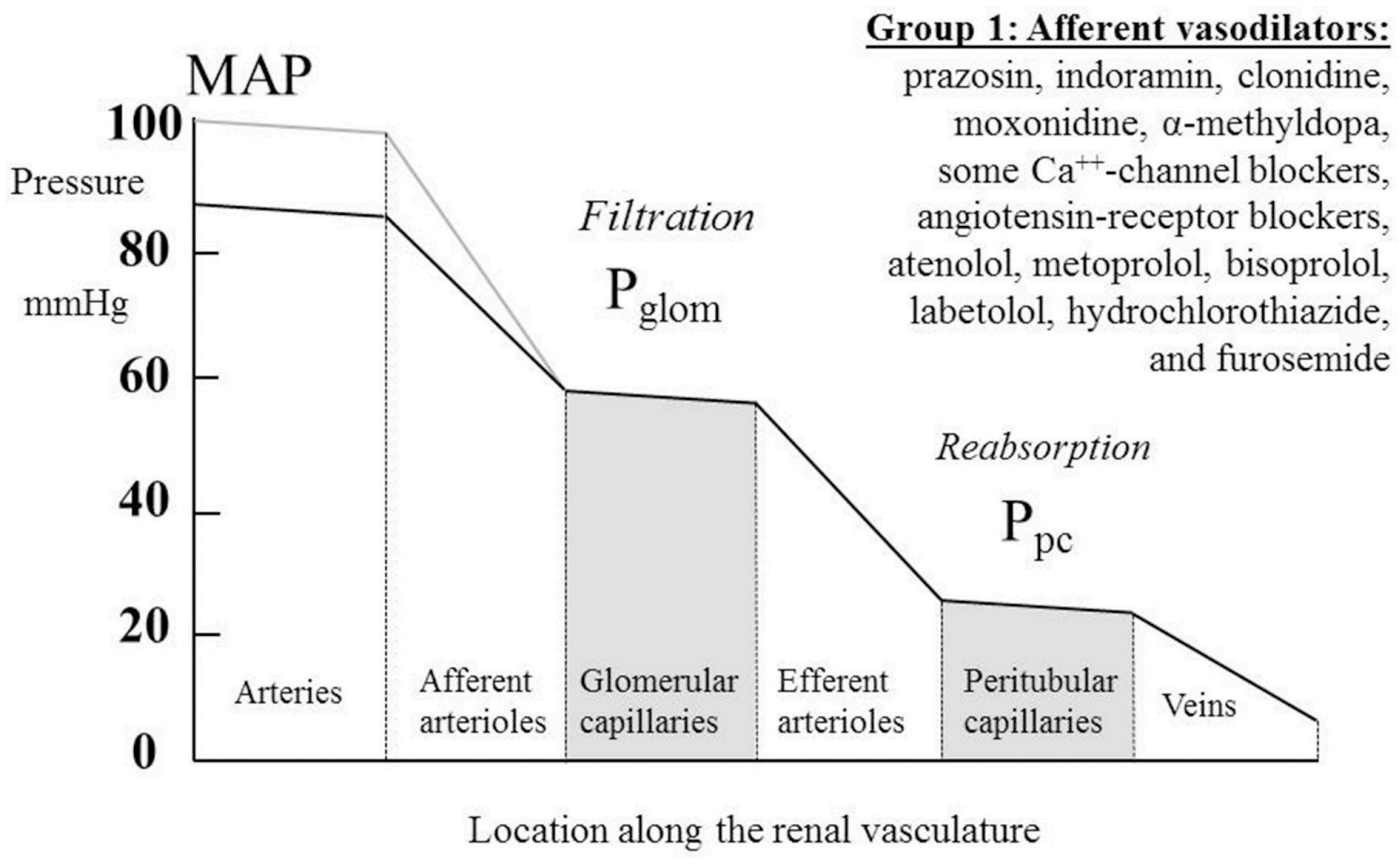
**The actions on the hydrostatic pressure profile along the renal vasculature of drugs that primarily dilate the afferent arterioles and leave glomerular filtration rate, glomerular capillary pressure (P_glom_), and pressures downstream of the glomerular capillaries unchanged**. MAP, mean arterial pressure; P_pc_, peritubular capillary pressure.

Table 1 summarizes renal hemodynamic measurements of drugs classically regarded as “vasodilators.” This table includes information for α_1_-blockers (prazosin, indoramin), an α_2_-agonist (clonidine), an imidazoline I_1_ receptor agonist (moxonidine), α-methyldopa, and Ca^++^-channel blockers (CCBs) (verapamil, diltiazem, manidipine, nicardipine, nitrendipine, isradipine, amlodipine, and nifedipine). Where measured, these studies invariably show a fall in RVR or R_A_, usually in the presence of an unchanged RBF; the exceptions to the latter were diltiazem, nifedipine, nitredipine, and isradipine, which showed an increase in RBF. With regard to GFR, most studies found no change, but a rise was noted in three studies involving nifedipine and amlodipine. The families of agents represented in Table 1 thus act primarily by achieving a sufficient degree of afferent arteriolar vasodilatation to permit the kidney to maintain blood flow and GFR despite a lower MAP. An unchanged GFR is consistent with an unchanged P_glom_ for the reasons discussed above. The associated profile of the renal vascular hydrostatic pressure resulting from this mechanism of drug action is depicted in Figure 3.

**Table 1:** **The change in human renal hemodynamics after long-term vasodilator treatment for hypertension**.

**Vasodilators**								
**Drug**	**References**	**RBF/RPF**	**P_glom_**	**GFR**	**FF**	**RVR**	**Duration (weeks)**	**Participant number**
Prazosin	Koshy et al., 1977	=		=			8	14
	Preston et al., 1979; Warren et al., 1981	=		=	=	R_A_: 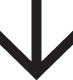	4	10 males^†^
						R_E_: =		
						R_V_: =		
	O'Connor et al., 1979	=		=	=		4	12 males
	Bauer et al., 1984b	=		=	=	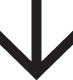	3–6	14
	Anderton et al., 1994	=		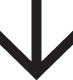			10	14
Indoramin	Bauer et al., 1984a	=		=	=	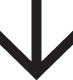	3–6	11 males
Clonidine	Cohen et al., 1979	=		=	=	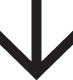	4	13
	Thananopavarn et al., 1982	=		=			12	16
	Golub et al., 1983	=		=			1	16
		=		=			12	As above
Moxonidine	Fauvel et al., 1998	=		=	=		4	20
α-Methyldopa	Weil et al., 1963	=		=			1–3	9
	Mohammed et al., 1968	=		=		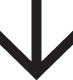	1	8
	Grabie et al., 1980	=		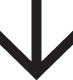			1	8
Verapamil	Leonetti et al., 1980			=			1.5	12
	Sorensen et al., 1985	=		=	=		6	11
	Katzman et al., 1990	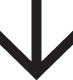		=		=	8	15
Diltiazem	Isshiki et al., 1987	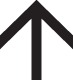	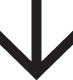	=	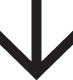	R_A_: 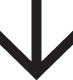	52	7
						R_E_: 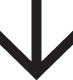		
	Sunderrajan et al., 1987	=		=	=	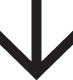	8	18
Manidipine	Ott et al., 2013	=	=	=		R_A_: 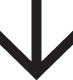	4	54
						R_E_: 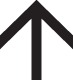		
Nicardipine	Smith et al., 1987	=		=		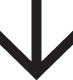	6	6
	Kimura et al., 1988			=			1	8
	Kimura et al., 1991a	=	=	=		R_A_: 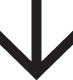	1	8
						R_E_: =		
Nitrendipine	Thananopavarn et al., 1984	=		=			2	10 males
	Scaglione et al., 1992	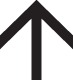		=	=	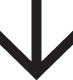	8	13
Isradipine	Persson et al., 1989	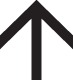		=	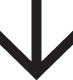		9	20 males
Amlodipine	Ranieri et al., 1994	=		=	=		12	18
	Inigo et al., 2001	=	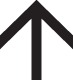	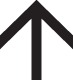	=	R_A_: 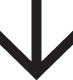 ^*^	6	17 (renal transplant recipients)
						R_E_: =		
	Delles et al., 2003	=	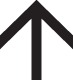	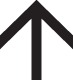	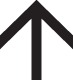	R_A_: 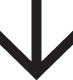	8	29
						R_E_: 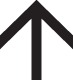		
	Ott et al., 2013	=	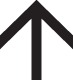	=		R_A_: 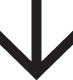	4	50
						R_E_: 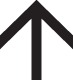		
Nifedipine	Olivari et al., 1979			=			3	27
	Guazzi et al., 1984			=			1	14
	Reams et al., 1988	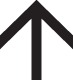		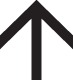	=	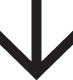	4	26

*Participants are individuals with essential hypertension unless otherwise specified. RBF/RPF, renal blood flow/renal plasma flow; GFR, glomerular filtration rate; FF, filtration fraction; RVR, renal vascular resistance; 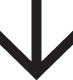, significant decrease from control; 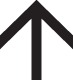, significant increase from control; =, no significant change from control; R_A_, afferent arteriolar resistance; R_E_, efferent arteriolar resistance*.

* *Discrepancy between reported result in the text and in the results table—mistake in decimal point placement in results table assumed (Inigo et al., 2001)*.

† *Identical data in two publications, one stating n = 10 and one n = 12*.

The suggestion of a raised GFR during treatment with certain CCBs prompted some researchers to estimate P_glom_ and the individual changes in R_A_ and R_E_ following chronic administration of these agents. The several findings with amlodipine, and single studies with two other drugs of this class, suggested that an unchanged or elevated R_E_ may be associated with a rise in P_glom_ and a rise in GFR. A single study (of diltiazem) found a marked reduction in both R_A_ and R_E_, accompanied by a rather distinctive fall in P_glom_ in the presence of unchanged GFR and a large rise in RBF (Isshiki et al., 1987). Non-human studies have suggested that a differential distribution of Ca^++^ channel subtypes between the afferent and efferent arterioles may be able to account for these effects (Hayashi et al., 2007), and have shown similar differential effects on the two vessel types. For example, acute administration of nifedipine to anesthetized dogs generated a 27% fall in R_A_ whilst leaving R_E_ unaffected (Heller and Horacek, 1990), whilst acute administration of diltiazem to spontaneously hypertensive rats reduced R_A_ by 40% and R_E_ by 18% (Isshiki et al., 1987).

### β-blockers

**Figure 4:**
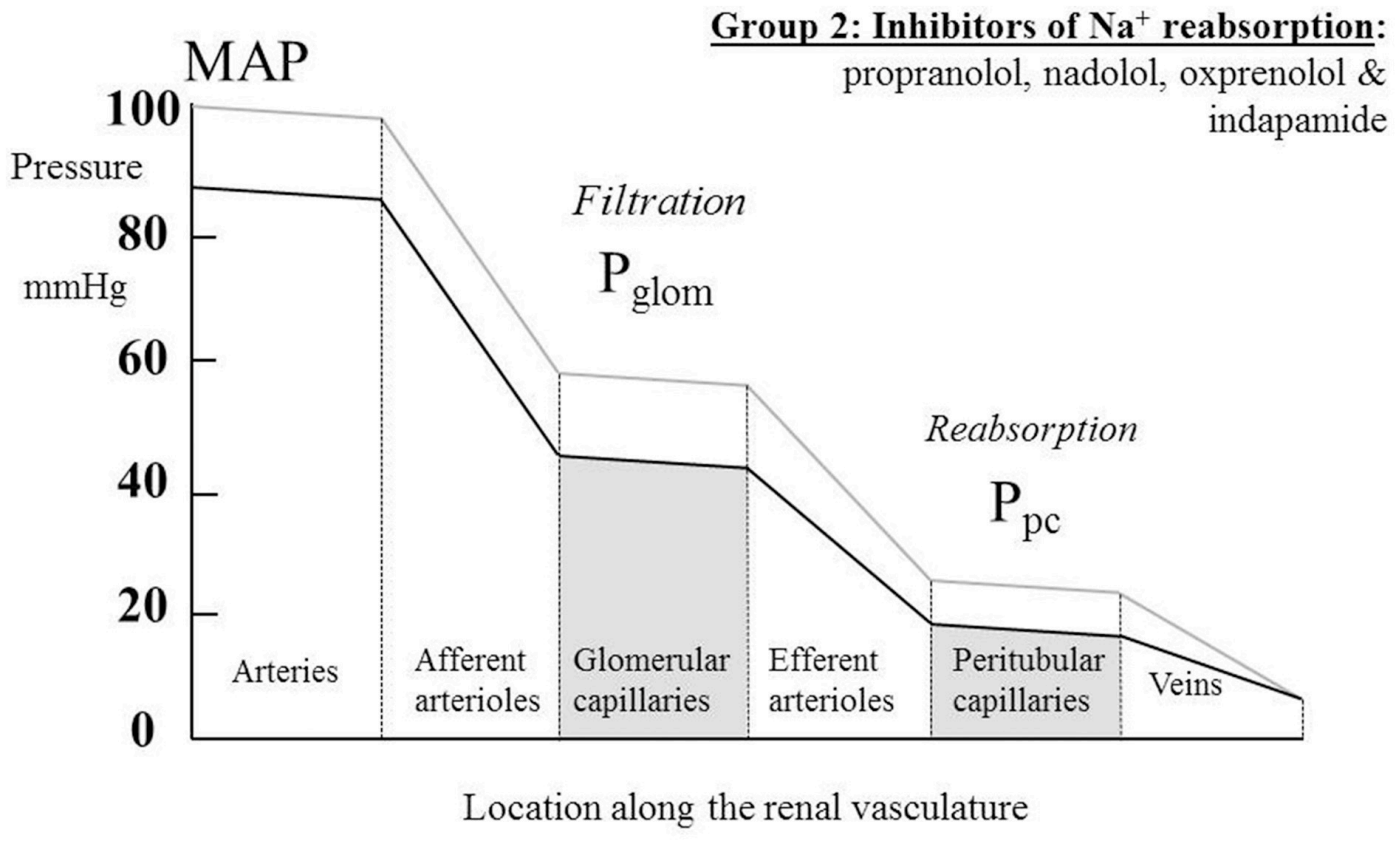
**The actions on the hydrostatic pressure profile along the renal vasculature of drugs that primarily redistribute the peritubular capillary pressure (P_pc_) component of the Starling forces across the tubular epithelium by inhibiting active solute uptake from the renal tubule**. MAP, mean arterial pressure; P_glom_, glomerular capillary pressure.

Table 2 summarizes human renal hemodynamic measurements obtained following administration, for at least a week, of β-blockers. Here we see a pattern of behavior for the non-selective agents propranolol and nadolol that act at both β_1_ and β_2_ receptors that is distinctively different from the agents in Table 1. RBF is commonly reduced by drug administration, RVR is unchanged or even elevated, and GFR never raised, but often found to be reduced. This remarkable finding is perhaps analogous to a reduction in renal tissue, and yet it remains consistent with a normal daily elimination of sodium and water. To reconcile these observations, it is clear that the proportion of sodium and water reabsorbed from a reduced glomerular filtrate must be reduced. A likely candidate mechanism would be inhibition of active components of tubular reabsorption. Unfortunately, we lack measurements in humans on long-term antihypertensive medication that would allow us to understand better the mechanism underlying this interesting effect of non-selective β-blockers. Figure 4 depicts a profile of renal vascular pressures consistent with the observations made in these studies.

**Table 2:** **The change in human renal hemodynamics after long-term beta-blocker administration**.

**β-Blockers**								
**Drug**	**References**	**RBF/RPF**	**P_glom_**	**GFR**	**FF**	**RVR**	**Duration (weeks)**	**Participant number**
Propranolol	Ibsen and Sederberg-Olsen, 1973			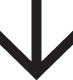			8–12	11
	Falch et al., 1978	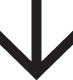					2	11 (males)
	Falch et al., 1979b	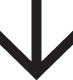					32	13
	O'Connor et al., 1979	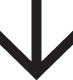		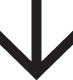	=		4	12 (males)
	Bauer and Brooks, 1979	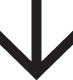		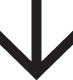	=		4	8 (normal subjects)
	Wilkinson et al., 1980			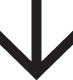			8	15
	Warren et al., 1981	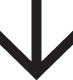		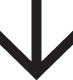	=	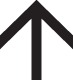	4	13
	Bauer, 1983	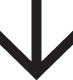		=	=	=	20–25	14 (males)
	Kimura et al., 1988			=			1	8
	Malini et al., 1982	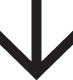		=		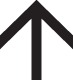	12	12
	O'Connor and Preston, 1982	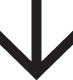		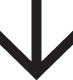	=	=	4	15 (males)
	Malini et al., 1984	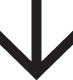		=		=	12	12
	van den Meiracker et al., 1989	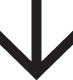		=		=	3	10 (males)
Nadolol	O'Connor et al., 1982	=		=	=	=^†^	6	10 (males)
	Textor et al., 1982	=		=		=^†^	8	15
	O'Callaghan et al., 1983	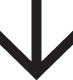		=		=	10	10 (elderly)
Oxprenolol	Bellini et al., 1982	=		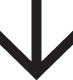			7	7
Atenolol	Wilkinson et al., 1980			=			8	15
	Dreslinski et al., 1982	=		=		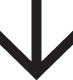	4	10
	Bellini et al., 1982	=		=			7	7
	O'Callaghan et al., 1983	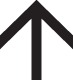		=		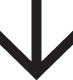	12	10 (elderly)
	van den Meiracker et al., 1989	=		=		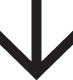	3	10 (males)
	Samuelsson et al., 1992	=		=	=	=^†^	4	17
	De Cesaris et al., 1993	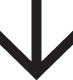		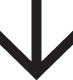		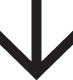	32	10 (hypertensive diabetics)
	Leeman et al., 1993	=		=		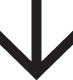	4	14
Metoprolol	Sugino et al., 1984	=		=	=	=^†^	5–7	9 (males)
Bisoprolol	Leeman et al., 1993	=		=		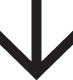	4	14
	Parrinello et al., 2009	=		=	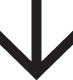	=^†^	52	36
Labetalol	Rasmussen and Nielsen, 1981			=			4–15	11
	Malini et al., 1982	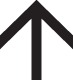		=		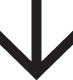	12	12

*Participants are individuals with essential hypertension unless otherwise specified. RBF/RPF, renal blood flow/renal plasma flow; GFR, glomerular filtration rate; FF, filtration fraction; RVR, renal vascular resistance; 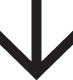, significant decrease from control; 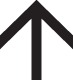, significant increase from control; =, no significant change from control*.

† *Apparently anomalous results, given that for individual subjects RVR = MAP/RBF, MAP falls significantly, and yet neither RBF nor RVR are found to change significantly; the discrepancy arises from the statistical distribution within the study group*.

The selective antagonists at β_1_ receptors, atenolol, metoprolol, and bisoprolol show a different behavior. Most studies of these agents in Table 2 show a contrasting fall in RVR. Findings for RBF are variable. In these respects we see a renal hemodynamic pattern similar to that of the α_1_-blocker prazosin, the prominent feature being an afferent arteriolar vasodilatation that is sufficiently great to permit the kidney to run at a lowered arterial pressure with indices of RBF and GFR changing little, if at all. The notion of a β_1_ antagonist having a primary vasodilatory action is an unfamiliar concept; SVR is unchanged or increased by β-blockers (Dreslinski et al., 1982; van den Meiracker et al., 1989).

Labetolol is thought of as having antagonist activity at α-receptors that exceeds its antagonist action at β-receptors. The observations shown in Table 2 for this drug are consistent with this picture, though the rise in RBF is a feature of only one other of the several studies in Tables 1, 2 of α-blockers and β-blockers, namely that by O'Callaghan et al. on atenolol (O'Callaghan et al., 1983). Atenolol and labetolol have characteristics that earn them a place in Figure 3 amongst a variety of drug families that have a primary vasodilatory action on the afferent arteriole.

### ACE inhibitors and AR blockers

Table 3 summarizes renal hemodynamic measurements following administration, for at least a week, of drugs inhibiting the renin-angiotensin system. Amongst the angiotensin converting enzyme (ACE) inhibitors, the usual pattern of response is for RVR to fall sufficiently to permit a rise in RBF in the presence of a lower MAP, and for this often to be associated with an increase in GFR. Unfortunately, estimates of P_glom_ are not available, but it is not unreasonable to assume, for reasons given above, that a rise in P_glom_ is driving the increase in GFR.

**Table 3:** **The change in human renal hemodynamics after long-term treatment for hypertension with drugs acting on the RAS**.

**ACE Inhibitors and AR Blockers**								
**Drug**	**References**	**RBF/RPF**	**P_glom_**	**GFR**	**FF**	**RVR**	**Duration (weeks)**	**Participant number**
Captopril	Ando et al., 1986	=				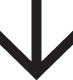	2	12
	Shionoiri et al., 1987	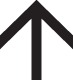		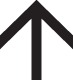	=	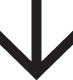	5–10 days	16
	“	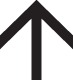		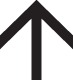	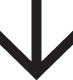	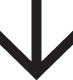	5–10 days	9 (malignant hypertension)
	Kimura et al., 1988			=			1	8
Enalapril	Simon et al., 1983	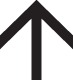		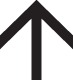			16	22
	Bauer, 1984; Bauer and Jones, 1984	=		=	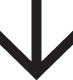	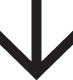	8	16
	“	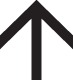		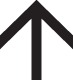	=	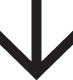	8	10 (subgroup with low GFR)
	Katzman et al., 1990	=		=	=	=	8	15
	De Cesaris et al., 1993	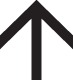		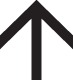		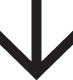	32	10 (hypertensive diabetics)
	Pechere-Bertschi et al., 1998	=		=	=	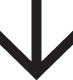	6	10
	De Rosa et al., 2002			=			156	20
Lisinopril	Dupont et al., 1987	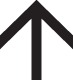		=		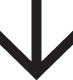	12	9
	Samuelsson et al., 1992	=		=	=	=^†^	4	17
	Degaute et al., 1992	=		=	=	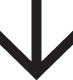	12	12
	Ranieri et al., 1994	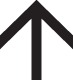		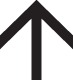	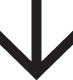		12	18
Losartan	Paterna et al., 2000	=		=	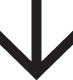	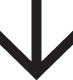	24	18
	Inigo et al., 2001	=	=	=	=	R_A_: 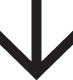 ^*^	6	17 (renal transplant recipients)
						R_E_: 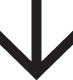 ^*^		
	De Rosa et al., 2002			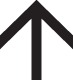			156	22
	Parrinello et al., 2009	=		=	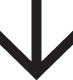	=^†^	52	36
Valsartan	Delles et al., 2003	=	=	=	=	R_A_: 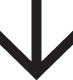	8	29
						R_E_: =		
Candesartan	Fridman et al., 2000	=		=	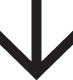	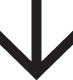	6	19

*Participants are individuals with essential hypertension unless otherwise specified. RBF/RPF, renal blood flow/renal plasma flow; GFR, glomerular filtration rate; FF, filtration fraction; RVR, renal vascular resistance; 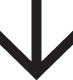, significant decrease from control; 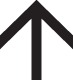, significant increase from control; =, no significant change from control*.

* *Discrepancy between reported result in the text and in the results table—mistake in decimal point placement in results table assumed (Inigo et al., 2001)*.

† *Apparently anomalous results, given that for individual subjects RVR = MAP/RBF, MAP falls significantly, and yet neither RBF nor RVR are found to change significantly; the discrepancy arises from the statistical distribution within the study group*.

The sparse data on angiotensin receptor (AR) blockers suggests a slightly different pattern similar to most of the drugs in the vasodilator group of Table 1, namely with a fall in RVR or R_A_, with RBF maintained unchanged. These drugs appear to be best depicted by Figure 3, but show some similarity with the ACE inhibitors with respect to a lowering of filtration fraction in some studies. For both kinds of agents in Table 3, there is a suggestion that filtration fraction may be decreased. In other words, the proportionate rise in RBF is tending to be even greater than that of GFR.

**Figure 5:**
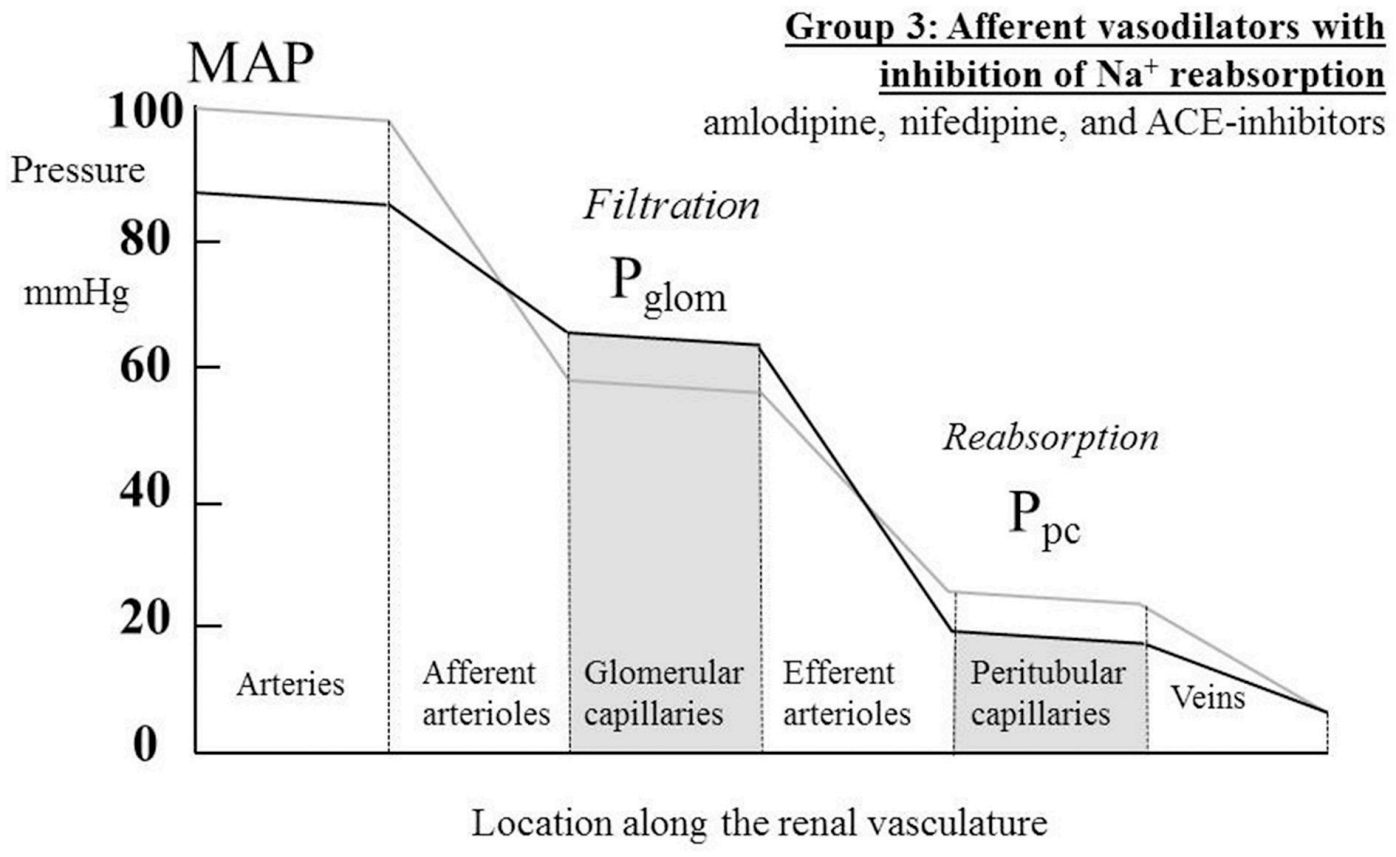
**The actions on the hydrostatic pressure profile along the renal vasculature of drugs that both dilate afferent arterioles and redistribute the peritubular capillary pressure (P_pc_) component of the Starling forces across the tubular epithelium**. These actions lead to a raised glomerular capillary pressure (P_glom_) and a greater pressure drop across the efferent arterioles, either due to efferent arteriolar constriction or an increased renal blood flow. MAP, mean arterial pressure.

For the increased GFR associated with the ACE-inhibitors to exist in the presence of a presumed unchanged sodium and water balance for the body as a whole, tubular reabsorption of sodium and water must be enhanced to counter the excess flux of filtrate. Given that these drugs are associated with an inhibition of the action of angiotensin II and aldosterone on sodium uptake by the proximal and distal tubules respectively, this finding suggests that the enhanced reabsorption is driven by a dominant increase in the passive Starling forces moving filtrate across into the peritubular capillaries. This would require a lower hydrostatic pressure in the peritubular capillaries during drug therapy, which would in turn require a greater pressure drop across the efferent arterioles, as blood passed from the glomerular into the peritubular capillaries. The different roles of the afferent and efferent arterioles in the action of this family of drugs are particularly striking. Figure 5 depicts a profile of renal vascular pressures consistent with these observations.

### Diuretics

Table 4 summarizes renal hemodynamic measurements following administration, for at least a week, of diuretic drugs. These drugs suffer from having a misleading family name; whilst acutely they induce a diuresis, familiar to all patients taking them, over a long period of administration the body remains in sodium and water balance without a net diuresis. The study of van Brummeln et al. over 9 months of therapy with hydrochlorothiazide in 10 hypertensives found that 24-h urinary sodium excretion remained unchanged, except at the 1-week point, where it was significantly lower than before treatment (Van Brummelen et al., 1979). In the same study a twice-daily regimen of furosemide administration was associated with a cycle of 6-h diureses following drug ingestion and 6-h periods of reduced urine output before the next dose. Overall there was no change in 24-h sodium excretion. The possibility that long-term diuretic therapy might lead to a prolonged increase in daily sodium flux, with a corresponding increased daily sodium intake to maintain equilibrium, appears to be excluded by this and another prolonged study (Roos et al., 1981). Similarly, maintenance of 24-h sodium excretion has been observed with other antihypertensives, including prazosin (Koshy et al., 1977) clonidine (Thananopavarn et al., 1982; Golub et al., 1983), moxonidine (Fauvel et al., 1998), propranolol (O'Connor and Preston, 1982), nadolol (O'Connor et al., 1982), amiloride (Matthesen et al., 2013), and spironolactone (Matthesen et al., 2013). Having said this, an increase in 24-h sodium excretion has been reported with both hydrochlorothiazide (O'Connor et al., 1981) and metoprolol (Sugino et al., 1984).

**Table 4:** **The change in human renal hemodynamics after long-term treatment for essential hypertension using diuretics**.

**Diuretics**								
**Drug**	**References**	**RBF/RPF**	**P_glom_**	**GFR**	**FF**	**RVR**	**Duration (weeks)**	**Participant number**
Hydrochlorothiazide	Van Brummelen et al., 1979	=		=	=	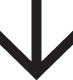	36	10 (males)
	O'Connor et al., 1981;Warren et al., 1981	=		=	=	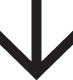	4	19 (males)
	Scaglione et al., 1992	=		=	=	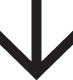	8	13
Chlorothiazide	Loon et al., 1989	=		=	=		4	9
Furosemide	Olshan et al., 1981;Warren et al., 1981	=		=	=	=^†^	4	12
Spironolactone	Falch et al., 1979a	=					12	10
	Matthesen et al., 2013			=			4	23
Furosemide and spironolactone	Loon et al., 1989	=		=	=		4	6
Amiloride	Matthesen et al., 2013			=			4	23
Indapamide	Pickkers et al., 1998	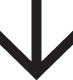		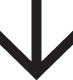	=		6	11

*RBF/RPF, renal blood flow/renal plasma flow; GFR, glomerular filtration rate; FF, filtration fraction; RVR, renal vascular resistance; 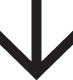, significant decrease from control; 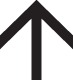, significant increase from control; =, no significant change from control*.

† *An apparently anomalous result, given that for individual subjects RVR = MAP/RBF, MAP falls significantly, and yet neither RBF nor RVR are found to change significantly; the discrepancy arises from the statistical distribution within the study group*.

The pattern of diuretic behavior seen in Table 4 is the same as for most of the “vasodilator” family seen in Table 1, with the exception of the thiazide-like drug indapamide. A fall in MAP is associated with no change in RBF or GFR. A fall in RVR, when calculated, reflects this maintenance of normal flows in the presence of a reduced perfusion pressure. Whilst we commonly associate the actions of the different families of diuretic drugs with inhibition of various different sodium reabsorption mechanisms in the renal tubule, here the dominant feature is their apparent vasodilatory potential in the afferent arteriole. Figure 3 best represents the evidence from these studies as an action of renal afferent vasculature leaving P_glom_ and P_pc_ unchanged. If a lowering of P_pc_ is required to overcome a degree of inhibition of active sodium reabsorption by these drugs, interestingly this seems not to translate back upstream into a lowering of P_glom_, given that GFR remains unaltered by these drugs. The exception to this pattern is indapamide. Its profile appears to be best described by Figure 4. It is noted below that this behavior is associated with a striking difference between indapamide and hydrochlorothiazide with regard to vasodilatory potential.

## Discussion

### Mechanisms of afferent arteriolar dilatation

We have observed remarkable similarities of action of several families of antihypertensive agents that fairly selectively dilate the afferent arteriole, thereby permitting the kidney to operate at a new, lower MAP, with indices of renal function downstream of the afferent arteriole left unchanged (Figure 3). The similarities of action that follow chronic administration should not necessarily be taken to imply similarities of mechanism. It is possible that some agents act as direct vasodilators, whilst others have indirect effects. Indeed, we have striking evidence of this being the case as we compare the diuretics with other drugs that reduce the pressure drop across the afferent arteriole.

**Figure 6:**
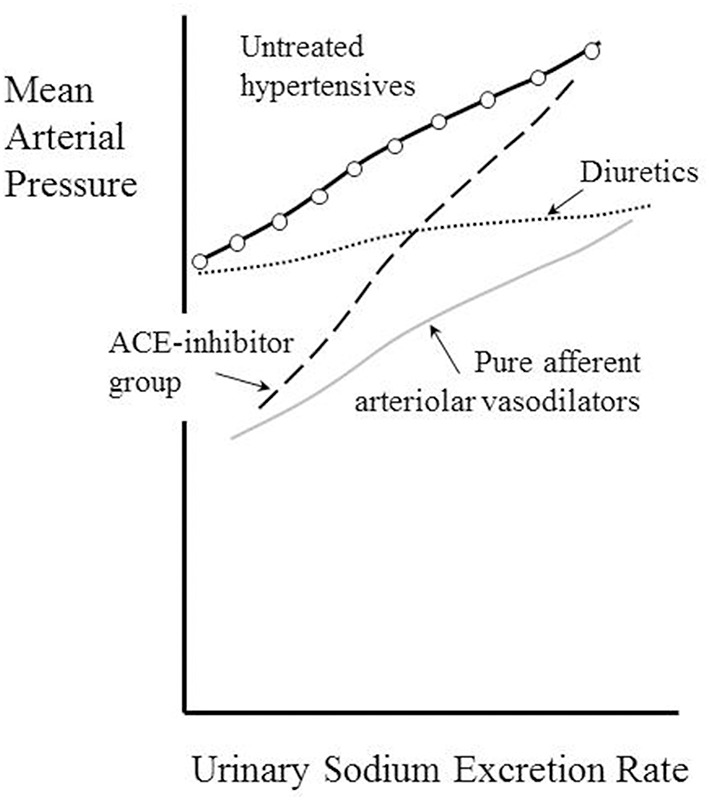
**Kimura's classification of antihypertensive drugs according to their effects of the pressure-natriuresis relationship**. Adapted from Dorrington and Pandit (2009).

A pure directly-acting dilator of the afferent arteriole might be expected to mimic the consequence of a surgical removal of a renal artery stenosis and thereby lead to a fall in MAP that does not depend upon the urinary sodium secretion, as demonstrated in patients with renovascular hypertension (Kimura et al., 1987). This phenomenon is depicted in Figure 6 as a parallel shift downwards of the pressure natriuresis curve, a type of effect that has been confirmed for nicardipine (Kimura et al., 1988).

A reduction in afferent arteriolar resistance that is independent of urinary sodium flux might be anticipated for drugs inhibiting vascular smooth muscle α-adrenoceptors, such as prazosin and indoramin, drugs reducing sympathetic efferent activity such as clonidine, moxonidine and α-methyldopa, and agents having a direct vasodilatory effect on vascular smooth muscle, such as Ca^++^-channel blockers. Similarly, given the known constrictor action on afferent arterioles of angiotensin II (Wilson, 1986), it is conceivable that a main long-term action of AR blockers is dilatation of the afferent arterioles. The data of Table 2 suggest that this effect dominates over the inhibition of the direct renal tubular actions of angiotensin II (Geibel et al., 1990) and its action via stimulation of adrenal aldosterone release (Campbell et al., 1974). That the selective β-blocker atenolol has a mainly vasodilatory action on the afferent arterioles may be an indirect effect, secondary to inhibition of renin release (Wilkinson et al., 1980; Dreslinski et al., 1982; van den Meiracker et al., 1989) and a resultant decrease in angiotensin levels.

**Figure 7:**
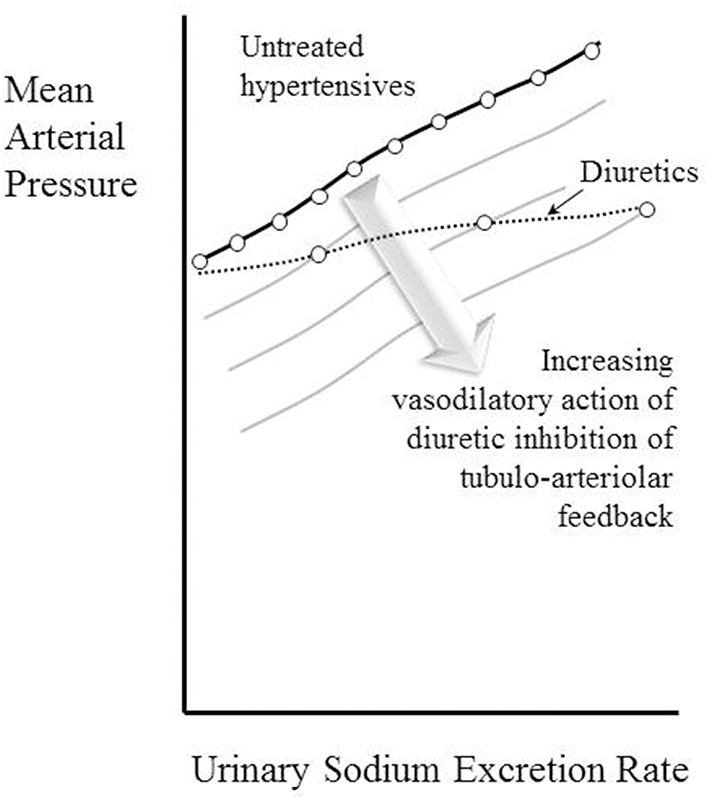
**Depiction of the hypothesis that increasing vasodilatory action of diuretics inhibiting tubulo-arteriolar feedback can account for the decrease in gradient of the pressure-natriuresis line brought about by these drugs**. A constant degree of afferent arteriolar dilatation leads to a parallel downward shift of the pressure-natriuresis line (as shown in Figure 6). The stack of pressure-natriuresis lines depicted here for diuretics arises because the degree of afferent arteriolar dilatation is hypothesized to depend upon inhibition tubulo-arteriolar feedback, which in turn depends upon the sodium excretion rate.

The observation that the chronic action of diuretics is primarily as afferent arteriolar dilators (Table 4, Figure 3) is perhaps most surprising of all, given that their main associations are with tubular sodium uptake mechanisms. Relevant here may be the known inhibition of *macular densa* cell function in the tubulo-arteriolar (also known as tubulo-glomerular) feedback mechanism by furosemide (Schnermann, 2003; Orlov and Mongin, 2007) and the recent elucidation of a more distal tubulo-arteriolar feedback mechanism that can be affected by inhibitors of the epithelial sodium channel (ENaC), such as amiloride (Ren et al., 2013; Wang et al., 2015). If the main long-term action of diuretics were via their inhibition of tubulo-arteriolar feedback, we would anticipate that the effect would depend upon sodium flux, with greater dilatation being evident at higher levels of urinary sodium excretion rate. This appears to be precisely the phenomenon that is observed with a thiazide, as shown in Figure 2 (Saito and Kimura, 1996). In Figure 7 we illustrate the hypothesis that increasing inhibition of tubulo-arteriolar feedback with increasing sodium excretion may underlie the reduction in gradient of the pressure-natriuresis line brought about by a diuretic. It is of interest that a review on thiazides in 2004 started with the statement that “despite their extensive use, the mechanism by which these drugs lower blood pressure in the long term remains unknown” and concluded that the most likely mechanism was via “vasodilator actions” (Hughes, 2004). This view remains unchanged (Duarte and Cooper-DeHoff, 2010). Interestingly, a direct vasodilatory action on human systemic (forearm) vessels of hydrochlorothiazide has been found to be mediated via potassium channel activation and not by inhibition of vascular membrane Na-Cl cotransport (Pickkers et al., 1998). The direct comparison in this study with the thiazide-like drug indapamide showed the latter to have no vasodilatory effect in the human forearm, which may help explain why the action of indapamide in hypertension appears to be mainly via inhibition of tubular reabsorption, analogous to the action of the non-selective β-blockers, as depicted in Figure 4.

Relevant to the consideration that inhibitors of membrane sodium transport may have a substantial direct or indirect effect on the vasculature is the recent review by Kurtz and colleagues regarding Mendelian forms of salt-dependent hypertension (Kurtz et al., 2015). These authors argue that, although it has been widely assumed that the effects on blood pressure of congenital renal abnormalities of sodium transport can be attributed to their actions on extracellular fluid volume, a strong case can be made for an alternative “vasodysfunction” influencing MAP. These authors take the view that the pathophysiological action is via changes in SVR; they do not consider the possibility that an effect on the *renal* vasculature may provide the link between such vasodysfunction and abnormal MAP, a possibility that we hope this current review might encourage researchers to explore.

### Mechanism for reduction of pressure throughout the renal vasculature

The non-selective β-blocking drugs (propranolol, nadolol, and oxprenolol) appear unique in achieving their hypotensive action in association with a fall in GFR. They are also distinctive in reducing RBF and, in some studies, elevating RVR. We argue above, in relation to Figure 4, that the pressure profile along the renal vasculature is likely to be lowered throughout by the drugs. It appears that the dominant action is an inhibition of solute uptake from the tubule leading to a rebalancing of Starling forces and a lowering of P_pc_. These distinctive renal hemodynamics focus interest on the β_2_-adrenoceptors in the kidney. It is known that different genotypes of the β_2_-adrenoceptors are associated with different MAP (Snyder et al., 2006) and may be associated with hypertension (Kotanko et al., 1997). One hypothesis for this is that β_2_ stimulation in the renal tubule enhances ENaC function (as it does in the lung alveolar epithelium) and enhances active sodium re-absorption (Snyder et al., 2005). Another is that the β_2_ receptor is involved in aldosterone secretion from adrenal glomerulosa cells (Pojoga et al., 2006).

We noted above that the effect of non-selective β-blocking drugs is somewhat analogous to a reduction in renal tissue, despite preservation of normal daily elimination of sodium and water. One possibility that we have not entertained so far is an influence of drugs on glomerular mesangial cells. These smooth-muscle-like cells in the glomerulus have the potential to reduce the filtration coefficient K and modify blood flow to the glomerulus (Mene et al., 1989). A wide variety of signaling systems has been identified in these cells, including those affected by β-adrenergic agents. One study has identified a role for β_2_ stimulation with apoptotic reduction in cell numbers (Muhl et al., 1996). We should bear in mind, as we interpret the results of the renal function tests for all classes of drug, that the association between GFR and P_glom_ expressed in Equation (3) involves a permeability coefficient that is itself under physiological and possibly pharmacological control.

Kimura et al. examined the effect of sodium excretion rate on the antihypertensive action of propranolol in hypertensive patients. In relation to the way we have plotted the pressure-natriuresis curves in Figure 6, they observed a non-significant increase in gradient of the line together with a downward shift of the intercept at zero excretion of 20 mmHg (Kimura et al., 1988). This suggests that propranolol may have an action on this relationship that is the opposite of that seen with diuretics, namely producing a steeper, rather than shallower, pressure-natriuresis line than that seen before treatment.

Insofar as a non-selective β-blocking drug like propranolol will have some β_1_-blocking action like that shown by the selective agents such as atenolol, we may anticipate that some afferent arteriolar vasodilatory action will contribute to the lowering of MAP in Figure 4 in an analogous manner to that seen in Figure 3.

### Mechanisms for increasing the pressure drop across the efferent arteriole

Figure 5 depicts a hydrostatic pressure profile along the renal vasculature that is consistent with a lowered MAP in the presence of a raised GFR. In the case of some Ca^++^-channel blockers (amlodipine and manidipine) an increase in R_E_ has been measured in the presence of an unchanged RBF (Table 1). The responses of the efferent arterioles to these agents are clearly very different from the afferent arterioles (Ito and Abe, 1997). In the case of ACE inhibitors the raised GFR is associated with an increase in RBF (Table 3); in this case we may presume that an increase in pressure drop across the efferent arteriole may occur even if R_E_ is not raised. Unfortunately, no individual measurements of R_A_ and R_E_ are reported for ACE inhibitors.

A question arises as to why the AR blockers appear to have a somewhat different action on the renal vascular pressure profile from the ACE inhibitors, given that both agents diminish the action of angiotensin II. At least three factors seem material: the AR blockers act on only one of the two main angiotensin receptors, angiotensin II is formed in part by non-ACE pathways, and ACE is responsible for breaking down bradykinin—an endogenous dilator that augments renal blood flow (Epstein and Gums, 2005).

That the ACE-inhibitor family of drugs has a distinctive effect on renal function can be illustrated by Kimura's classification of diuretic action shown in Figure 6. In a study on captopril a marked steepening of the gradient of the pressure-natriuresis line was observed in hypertensives with an approximately fourfold change in slope (Kimura et al., 1988). This was analogous to the changes seen with captopril in dogs by Hall et al. (1980). We might speculate that increasing detachment of the kidney from control by the renin-angiotensin system makes the pressure-natriuresis relationship more like that of the unregulated isolated kidney: a filter requiring ever higher perfusion pressures to eliminate progressively higher sodium intakes (Kaloyanides et al., 1971).

### Future directions

Newer experimental approaches to treating hypertension involve the use of endothelin antagonists (e.g., bosentan), renin antagonists (e.g., aliskiren), and surgical renal denervation. So far, these studies have not made the long-term assessment of human renal hemodynamics a priority, so it is currently not possible to assess the renal changes brought about with any precision. Interestingly, a similar paucity of data is evident for the mode of action of agents that chronically elevate MAP such as non-steroidal anti-inflammatory drugs, corticosteroids and alcohol. Furthermore, older antihypertensive agents that had to some extent fallen out of fashion—such as indapamide (Peters et al., 2008), amiloride (Brown et al., 2016), hydrochlorothiazide (MacDonald et al., 2015), and spironolactone (Williams et al., 2015)—are being found in large trials to have surprising clinical utility, whilst we continue to remain relatively ignorant about their renal hemodynamic effects, and therefore their mode of antihypertensive action. It will be apparent to the reader that the current authors would very much like to see the collection of these sorts of data made a priority in future studies of such agents.

## Conclusions

Drugs proven to have sustained antihypertensive effects have profound actions on the kidney. These actions are of two main kinds: dilatation of the afferent arteriole, and inhibition of solute uptake from the renal tubule. Both actions change the hydrostatic pressure profile along the renal blood vessels, the first by changing vascular tone and the second by rebalancing the contributions to the Starling forces made by hydrostatic and osmotic pressures driving reabsorption. Some drugs show both modes of action. The actions of antihypertensive drugs elsewhere on the body are sometimes beneficial and often adverse; these actions may be less relevant to their effects on lowering blood pressure in the long-term, but much attention is paid to these incidental effects in the literature.

The literature also reveals striking and often counterintuitive findings, including that diuretics act as afferent vasodilators, as do selective β-blockers and AR-blockers. It is remarkable that the drugs most clearly identifiable as primarily reducing active sodium reabsorption are those having β_2_ inhibitory activity. It remains a challenge to physiology to interpret the divergent effects of drugs on afferent and efferent arterioles seen with some Ca^++^-channel blockers and the ACE inhibitors.

Though clearly sufficient to permit reasonably firm conclusions about common renal mechanisms of action of antihypertensive drugs, studies in humans of the renal hemodynamic effects over extended periods would benefit from being more rigorous and numerous. Many have been motivated by an attempt to display “favorable” renal effects, by which is usually meant no reduction in GFR or RBF (Textor et al., 1982; Bauer, 1984; Sugino et al., 1984; Dupont et al., 1987; Smith et al., 1987; Reams et al., 1988; Degaute et al., 1992; De Rosa et al., 2002), when it is clear that neither of these is a prerequisite for clinical efficacy. Furthermore, some studies misleadingly identify a fall in RVR as contributing to an antihypertensive effect only in proportion to the contribution this fall makes to the decrease in overall SVR (Koshy et al., 1977; Preston et al., 1979; Warren et al., 1981; Thananopavarn et al., 1982; Bauer, 1984; Dupont et al., 1987; Smith et al., 1987; van den Meiracker et al., 1989; Fridman et al., 2000). The difficulty in humans of identifying the separate components of the RVR, R_A_, R_E_, and R_V_, has already been discussed.

The distinctive differences between families of drugs with regard to their effect on the pressure-natriuresis relationship are clearly of considerable clinical significance. Information about the interaction of dietary sodium intake and antihypertensive medication would be valuable to physicians. Given the huge importance of antihypertensive medication, future studies should seek to determine with greater clarity how these drugs work. In order to do this effectively, we suggest the focus will by necessity be on the kidney.

## Author contributions

HD, MF, and KD have substantially contributed to the conception of the work, research in the literature, and the writing of the manuscript.

## Funding

Dr. Dorrington is supported by the Dunhill Medical Trust (grant R178/1110). Dr. Frise is the recipient of a British Heart Foundation Clinical Research Training Fellowship (FS/14/48/30828).

### Conflict of interest statement

The authors declare that the research was conducted in the absence of any commercial or financial relationships that could be construed as a potential conflict of interest.
